# Interaction effects of testosterone and platelet-to-lymphocyte ratio on osteoporosis in U.S. adults: Insights from the NHANES 2011 to 2016 cohort

**DOI:** 10.1097/MD.0000000000048306

**Published:** 2026-04-24

**Authors:** Chenglu Tao, Yingqi Liu, Huarui Shen, Ting Li, Yue He

**Affiliations:** a Chongqing Municipality Clinical Research Center for Endocrinology and Metabolic Diseases, Chongqing University Three Gorges Hospital, Chongqing, China; b Key Laboratory of Luminescence Analysis and Molecular Sensing, School of Materials and Energy, Southwest University, Chongqing, China; c Southwest University Hospital, Chongqing, China; d Department of Orthopedics, The Affiliated Traditional Chinese Medicine Hospital, Southwest Medical University, Luzhou, Sichuan, China; e School of Pharmacy, Southwest Medical University, Luzhou, Sichuan, China; f Sichuan Provincial Bayi Rehabilitation Center (Sichuan Provincial Rehabilitation Hospital), Chengdu, Sichuan, China.

**Keywords:** inflammation, NHANES, osteoporosis, platelet-to-lymphocyte ratio, testosterone

## Abstract

Testosterone (TST) deficiency and systemic inflammation may both contribute to osteoporosis, yet their joint effects remain underexplored. The platelet-to-lymphocyte ratio (PLR), a readily measurable marker of inflammation, may interact with TST to influence osteoporosis risk. To assess the independent and interactive associations between serum TST and PLR with osteoporosis in U.S. adults using National Health and Nutrition Examination Survey 2011 to 2016 data. We analyzed adults aged ≥20 years with complete data on bone mineral density, TST, and PLR. Osteoporosis was defined per World Health Organization criteria based on dual-energy X-ray absorptiometry bone mineral density. Low TST in men was defined as <300 ng/dL; PLR quartiles were derived from survey data, and cutoff points identified using restricted cubic spline analysis. Weighted logistic regression, restricted cubic spline modeling, and multiplicative interaction analysis were performed. Sensitivity analysis was conducted in participants ≥50 years. Weighted mean age was 39.5 years; 33.2% were male. Osteoporosis prevalence was 5.9%. Higher PLR showed a nonlinear positive association with osteoporosis risk (*P* for nonlinearity < .05), characterized by a steep increase in risk above the upper quartile. Lower TST was associated with increased odds of osteoporosis in men. A significant multiplicative interaction between low TST and high PLR was observed (*P*-interaction < .05). Results were consistent in adults ≥50 years. In U.S. adults, elevated PLR and lower TST were independently associated with greater odds of osteoporosis, with evidence of synergistic interaction. Prospective studies are warranted to validate these findings.

## 1. Introduction

Osteoporosis represents a metabolic bone disease marked by diminished bone mineral content and deterioration of bone tissue microstructure, elevating susceptibility to low-trauma fractures.^[1]^ Among aging males, heightened vulnerability to this condition stems primarily from senescence-related physiological alterations. Epidemiological data from the World Health Organization indicates a prevalence of 6% in men versus 21% in women above 50 years, accounting for more than 9 million fragility fractures each year.^[2,3]^ Clinical observations reveal that male osteoporosis patients frequently experience worse fracture outcomes than their female counterparts.^[4]^

As a principal male sex hormone, testosterone (TST) plays a pivotal role in maintaining musculoskeletal health through its effects on osseous tissue mineralization, muscular development, and articular cartilage maintenance.^[5,6]^ The skeletal system reciprocally influences TST production via osteocalcin, an osteoblast-derived endocrine factor.^[7]^ The age-related decline in TST is a key determinant of bone loss and fragility fractures in men.^[8–11]^

The platelet-to-lymphocyte ratio (PLR) has emerged as a contemporary biomarker of subclinical inflammatory status and correlates with diverse pathological states.^[12]^ Concurrently, systemic inflammation has been implicated in the pathogenesis of osteoporosis. While TST deficiency and inflammation are individually linked to bone loss, their potential interaction remains underexplored.^[13,14]^ Interestingly, TST demonstrates immunomodulatory capacity,^[15,16]^ whereas androgen deficiency correlates with heightened inflammatory mediators.^[17]^

This investigation employs National Health and Nutrition Examination Survey (NHANES) 2011 to 2016 datasets to examine potential interrelationships between inflammatory markers (particularly PLR), androgen levels, and osteoporosis development in aging males. The study seeks to clarify these associations and their clinical implications for preventive strategies and therapeutic management of bone metabolic disorders.

## 2. Methods

### 2.1. Study population and design

We used data from the NHANES cycles 2011 to 2016, conducted by the National Center for Health Statistics. NHANES employs a stratified, multistage probability sampling design to represent the U.S. civilian, noninstitutionalized population. Participants undergo structured interviews, standardized physical examinations, and laboratory tests.

**Figure 1.**
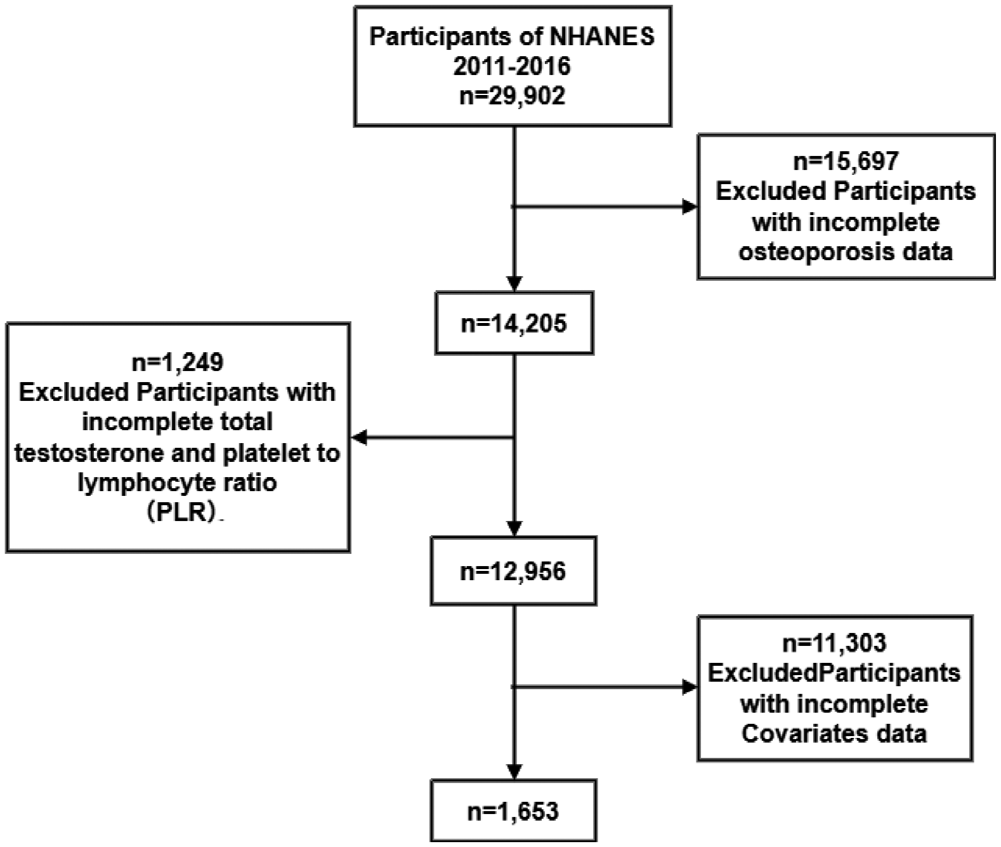
Flow chart of the study participants selection process. A total of 29,902 participants from the National Health and Nutrition Examination Survey (NHANES) cycles between 2011 and 2016 were initially included. After excluding 15,697 participants with incomplete osteoporosis data, 14,205 remained. Subsequently, 1249 participants were excluded due to missing total testosterone and platelet-to-lymphocyte ratio (PLR) data, leaving 12,956. Finally, 11,303 participants were excluded for incomplete covariates data, resulting in 1653 participants ultimately included in the analysis.

From the 29,902 NHANES participants in 2011 to 2016, we included adults aged ≥ 20 years who had valid femoral neck bone mineral density (BMD) measurements by dual-energy X-ray absorptiometry, serum TST measurements, and complete platelet and lymphocyte counts. Participants missing any covariates required for multivariable adjustment were excluded. The final analytic sample was 1653 individuals (Fig. 1). The study was approved by the National Center for Health Statistics Research Ethics Review Board, and all participants provided informed consent.

### 2.2. Definition of osteoporosis

BMD was measured at the femoral neck using a Hologic QDR 4500A fan-beam densitometer. Osteoporosis was defined according to the World Health Organization (WHO) criterion: *T*-score ≤–2.5 at the femoral neck, based on the reference range of young adults aged 20 to 29 years.^[18]^ Osteoporosis is typically diagnosed when BMD values in the total hip, femoral neck, or lumbar spine are 2.5 standard deviations or more below the mean BMD of young adults.^[19,20]^ We acknowledge that WHO criteria have been primarily validated in adults aged ≥50 years; therefore, we conducted a sensitivity analysis restricted to this age group.

### 2.3. PLR assessment

Platelet and lymphocyte counts were obtained from complete blood counts using a Beckman Coulter automated analyzer. PLR was calculated as platelet count divided by lymphocyte count (both in ×10^9^/L). Participants were divided into quartiles based on weighted population distribution (*Q*1–*Q*4), with *Q*1 as the reference category. cutoff points for further nonlinear analysis were identified using restricted cubic spline (RCS) modeling.

### 2.4. TST assessment

Total serum TST was measured from morning fasting blood samples using isotope dilution liquid chromatography tandem mass spectrometry. In men, low TST was defined as <300 ng/dL, based on Endocrine Society clinical guidelines. For analyses including both sexes, TST was modeled as a continuous variable and by sex-specific quartiles. Threshold points for nonlinear modeling were determined using RCS analysis.

### 2.5. Covariates

This analysis incorporated potential confounding factors that could influence the association between TST and osteoporosis. The covariates included demographic characteristics (gender, age, race, and marital status), socioeconomic factors (education level and poverty-to-income ratio), lifestyle factors (alcohol consumption), and health conditions (hypertension, diabetes, hyperlipidemia, and body mass index [BMI]). Race/ethnicity was categorized into 5 groups: Mexican American, non-Hispanic White, non-Hispanic Black, other Hispanic, and other races (including multiple races). Participants were divided into 3 groups based on the family poverty-to-income ratio: ≤1.0, 1.0 to 3.0, and >3.0. Education level was categorized into 5 groups: <9th grade, 9th–11th grade, high school graduate, some college or associate degree, and college graduate or above. Diabetes was defined as fasting plasma glucose ≥7.0 mmol/L, glycated hemoglobin (hemoglobin A1c) ≥6.5%, or a previous diagnosis of diabetes by a doctor.^[21]^ Hypertension was defined as systolic blood pressure or diastolic blood pressure ≥140 or 90 mm Hg, respectively, or a previous diagnosis of hypertension by a doctor, with blood pressure values being the average of 3 measurements taken on different dates.^[22]^ Hypercholesterolemia was defined as a previous diagnosis of hypercholesterolemia by a doctor. BMI was calculated by dividing a person’s weight (in kilograms) by the square of their height (in meters), presented as kg/m^2^.^[23]^ Descriptions of each variable can be found at Centers for Disease Control and Prevention NHANES.

### 2.6. Statistical analysis

In descriptive statistics, categorical variables were presented as frequencies and percentages, with differences between groups compared using the Chi-square test. Continuous variables with a normal distribution were presented as means and standard deviations, and differences between groups were assessed using the independent samples *t*-test. For continuous variables with a non-normal distribution, medians and quartiles were used, with differences tested via the Mann–Whitney *U* test. Univariate logistic regression analysis examined the association between all variables and osteoporosis.

Variables with a *P*-value <.05 were included in multivariate logistic regression analysis, resulting in 3 models: Model 1, unadjusted; Model 2, adjusted for age and gender; and Model 3, further adjusted for race, marital status, education level, family income ratio (poverty-to-income ratio), smoking, alcohol consumption, hypertension, hyperlipidemia, diabetes, and BMI. These models calculated the odds ratio (OR) with 95% confidence intervals (CI) and *P*-values for PLR and TST, assessing their independent correlation with osteoporosis across multiple subgroups.

Additionally, RCS analysis was employed to explore the nonlinear relationship and dose–response curve between PLR/TST and osteoporosis. An interaction term for PLR × TST was introduced in the regression model, with its significance (*P*-interaction < .05) evaluated through the likelihood ratio test. If significant, stratified analysis was conducted by grouping PLR into quartiles to examine differences in the effect of TST levels on osteoporosis at varying PLR levels. Sensitivity analysis restricted to participants aged ≥50 years was performed to test the robustness of findings.

All statistical analyses were performed using R (The R Foundation for Statistical Computing, R Core Team) version 4.4.2 and SPSS Statistics version 27 (IBM Corp., Armonk). A two-sided *P* < .05 was considered statistically significant.

## 3. Results

### 3.1. Baseline characteristics

Among 1653 eligible participants from NHANES 2011 to 2016 (selection flow in Fig. 1), the weighted mean age was 39.5 years; 33.2% were male. The weighted prevalence of osteoporosis was 5.9% (n = 97). Compared with those without osteoporosis, participants with osteoporosis were more likely to be female, older, have lower BMI, lower educational attainment, and comorbid hypertension or hyperlipidemia (Table 1). These descriptive findings align with the covariates used in subsequent weighted models.

**Table 1:** Baseline characteristics of the study population for osteoporosis.

Characteristics	Overall	Osteoporosis		*P*-value
		No	Yes	
n	1653	1556	97	
Age (yr)	39.52 ± 11.43	39.27 ± 11.40	43.57 ± 11.24	<.001
Gender, n (%)				<.001
Male	550 (33.27%)	522 (31.58%)	28 (1.69%)	
Female	1103 (66.73%)	1034 (62.55%)	69 (4.17%)	
Race, n (%)				<.001
Mexican American	277 (16.76%)	261 (15.79%)	16 (0.97%)	
Other Hispanic	177 (10.70%)	168 (10.16%)	9 (0.54%)	
Non-Hispanic White	408 (24.68%)	388 (23.47%)	20 (1.21%)	
Non-Hispanic Black	389 (23.53%)	367 (22.20%)	22 (1.33%)	
Other races	402 (24.32%)	372 (22.50%)	30 (1.81%)	
Education, n (%)				<.001
<9th grade	156 (9.44%)	140 (8.47%)	16 (0.97%)	
9–11th grade	192 (11.62%)	182 (11.01%)	10 (0.61%)	
High school graduate	345 (20.87%)	327 (19.78%)	18 (1.09%)	
Some college or AA degree	523 (31.64%)	498 (30.13%)	25 (1.51%)	
College graduate or above	437 (26.44%)	409 (24.74%)	28 (1.69%)	
Marital status, n (%)				<.001
Married	904 (54.69%)	850 (51.42%)	54 (3.27%)	
Widowed	26 (1.57%)	22 (1.33%)	4 (0.24%)	
Divorced	118 (7.14%)	114 (6.90%)	4 (0.24%)	
Separated	56 (3.39%)	52 (3.14%)	4 (0.24%)	
Never married	412 (24.92%)	389 (23.53%)	23 (1.39%)	
Living with partner	137 (8.29%)	129 (7.80%)	8 (0.48%)	
PIR, n (%)				<.001
<1	446 (26.98%)	422 (25.53%)	24 (1.45%)	
1–3	662 (40.05%)	621 (37.57%)	41 (2.48%)	
>3	545 (32.97%)	513 (31.03%)	32 (1.94%)	
Alcohol use, n (%)				<.001
Yes	755 (45.67%)	711 (43.01%)	44 (2.66%)	
No	898 (54.33%)	845 (51.12%)	53 (3.21%)	
Hypertension, n (%)				<.001
Yes	364 (22.02%)	338 (20.45%)	26 (1.57%)	
No	1289 (77.98%)	1218 (73.68%)	71 (4.30%)	
Hyperlipidemia, n (%)				<.001
Yes	385 (23.29%)	356 (21.54%)	29 (1.75%)	
No	1268 (76.71%)	1200 (72.60%)	68 (4.11%)	
Diabetes, n (%)				<.001
Yes	167 (10.10%)	154 (9.32%)	13 (0.79%)	
No	1486 (89.90%)	1402 (84.82%)	84 (5.08%)	
BMI, n (%)				<.001
Underweight	60 (3.63%)	58 (3.51%)	2 (0.12%)	
Normal weight	471 (28.49%)	452 (27.34%)	19 (1.15%)	
Overweight	467 (28.25%)	429 (25.95%)	38 (2.30%)	
Obesity	655 (39.62%)	617 (37.32%)	38 (2.30%)	

BMI = body mass index, PIR = poverty-to-income ratio.

### 3.2. Association between PLR and osteoporosis

**Figure 2.**
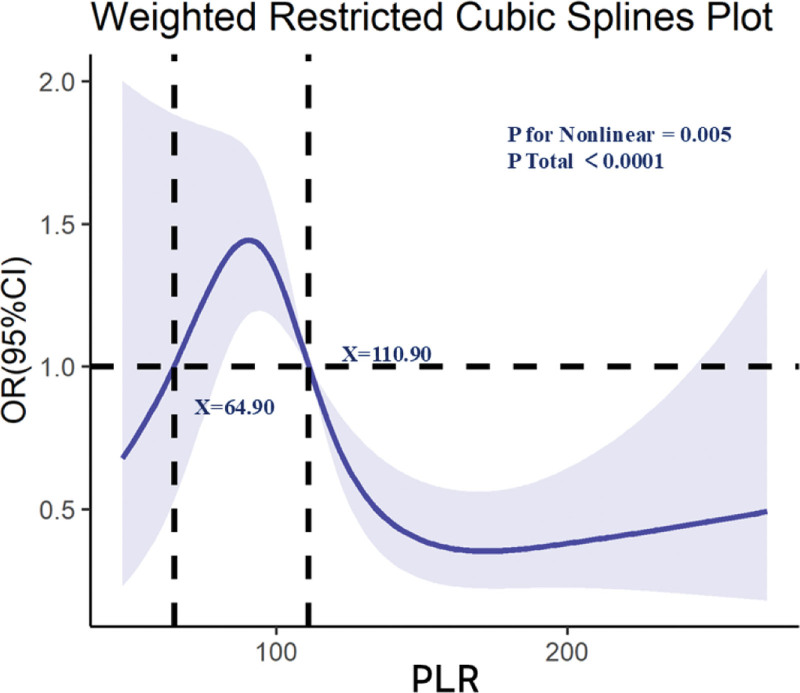
Dose–response relationship between PLR and osteoporosis risk (weighted restricted cubic splines). PLR = platelet-to-lymphocyte ratio.

In fully adjusted weighted logistic regression (Model 3, additionally controlling for age, sex, race/ethnicity, marital status, education, poverty-to-income ratio, smoking, alcohol use, hypertension, hyperlipidemia, diabetes, and BMI), participants in the highest PLR quartile (*Q*4) had significantly higher odds of osteoporosis than those in the lowest quartile (*Q*1) (OR = 3.58, 95% CI: 1.93–6.66; *P* < .001), with a significant trend across quartiles (*P* for trend < .001). No significant associations were observed for *Q*2 or *Q*3 relative to *Q*1 (Table 2). When modeled continuously, higher PLR was positively associated with osteoporosis risk; per 20-unit increase in PLR, the OR was 1.15 (95% CI: 1.04–1.27; *P* = .007; not shown). Weighted RCS analysis (Fig. 2) demonstrated a nonlinear positive association between PLR and osteoporosis (*P* for nonlinearity < .05), with a steeper risk increase above approximately the 75th percentile of PLR (~110.9) and a relatively flat slope below ~65, indicating little variation in risk at lower PLR values. PLR is a dimensionless ratio of platelet to lymphocyte counts.

**Table 2:** Weighted logistic regression analyses of association between platelet to lymphocyte ratio (PLR) and testosterone and osteoporosis.

	Model 1		Model 2		Model 3	
	OR 95% CI	*P* value	OR 95% CI	*P* value	OR 95% CI	*P* value
PLR						
Q1	Ref		Ref		Ref	
Q2	1.03 (0.49, 2.14)	.944	1.05 (0.50–2.19)	.899	1.10 (0.52–2.31)	.809
Q3	1.38 (0.70, 2.73)	.358	1.38 (0.70–2.74)	.356	1.47 (0.74–2.94)	.271
Q4	3.35 (1.83, 6.15)	<.001	3.27 (1.78–6.02)	<.001	3.58 (1.93–6.66)	<.001
*P* for trend		<.001		<.001		<.001
TST						
Normal	Ref		Ref		Ref	
Low	3.51 (2.29, 5.36)	<.001	3.11 (2.01–4.82)	<.001	3.11 (2.00–4.84)	<.001
*P* for trend		<.001		<.001		<.001

Model 1: no covariate adjustment. Model 2: additionally, adjusted for gender and age. Model 3: additionally, adjusted for race, marital status, education, poverty-income ratio, smoke, alcohol use, hypertension, hyperlipidemia, diabetes, and BMI.

BMI = body mass index, CI = confidence interval, OR = odds ratio.

### 3.3. Association between TST and osteoporosis

**Figure 3.**
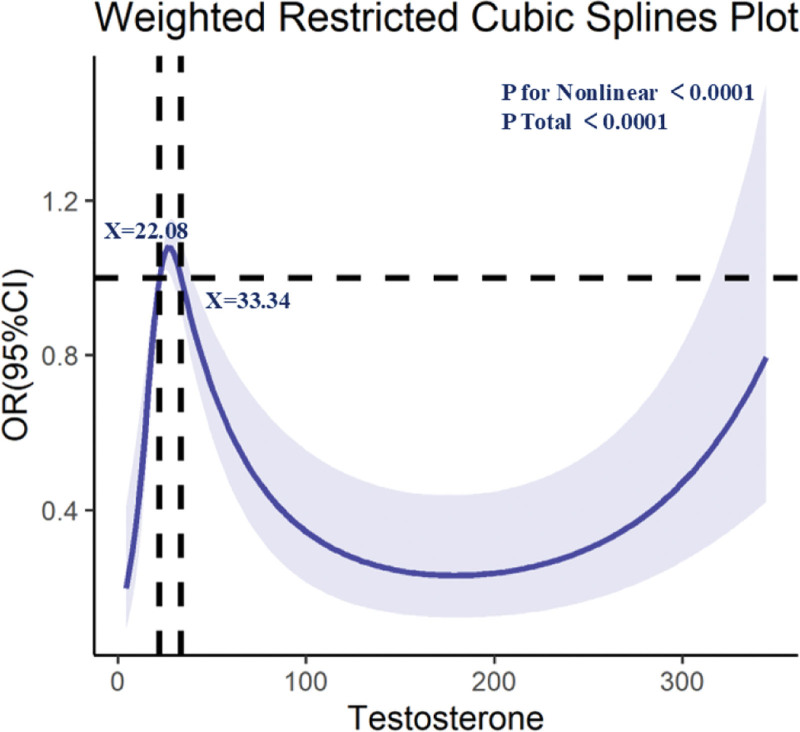
Dose–response relationship between testosterone (TST) and osteoporosis risk (weighted restricted cubic splines).

Among men, low TST (<300 ng/dL) was significantly associated with higher odds of osteoporosis (Model 3: OR = 3.11, 95% CI: 2.00–4.84; *P* < .001). When modeled continuously in men, higher TST was associated with lower osteoporosis risk (per 100 ng/dL increase, OR = 0.72; 95% CI: 0.58–0.89; *P* = .002; not shown). In women, no significant linear association was observed. RCS analyses suggested a nonlinear relationship between TST and osteoporosis (*P* for nonlinearity < .05). The spline shown in Figure 3 is based on the entire sample and reflects the combined distribution: osteoporosis risk declined sharply as TST rose from very low levels (<~22.08 ng/dL) into an intermediate range (~22.08–33.34 ng/dL), then plateaued with a slight upward inflection at higher concentrations. For clinical interpretation in men, the <300 ng/dL threshold remains the relevant benchmark.

### 3.4. Interaction between PLR and TST

A statistically significant multiplicative interaction was observed between PLR and TST in the weighted models (*P*-interaction < .05). In a joint categorization among men, those with both high PLR (*Q*4) and low TST had the highest odds of osteoporosis compared with the reference group (low PLR, *Q*1, and normal TST) (OR = 6.45, 95% CI: 3.02–13.78). Stratified spline patterns were consistent: when PLR exceeded ~110.9 and TST was in the low range (in the overall spline, ~<22.08 ng/dL; in men, <300 ng/dL), the predicted probability of osteoporosis increased sharply.

### 3.5. Subgroup analyses

**Figure 4.**
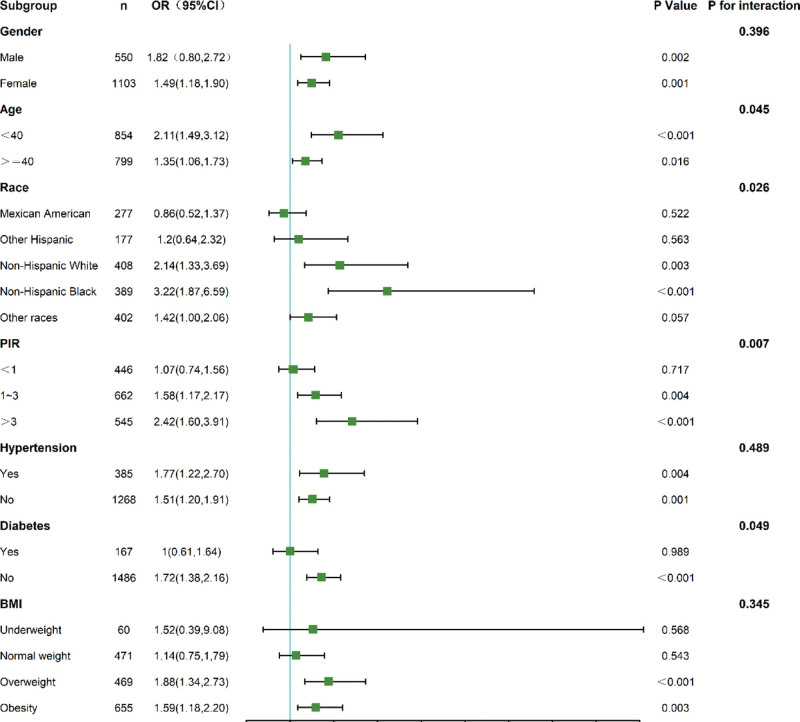
Subgroup analysis of platelet-to-lymphocyte ratio (PLR) and osteoporosis risk. Forest plot displaying ORs and 95% CIs for PLR-osteoporosis associations stratified by demographic and clinical factors. Restricted cubic splines adjusted for potential confounders. CIs = confidence intervals, ORs = odds ratios.

**Figure 5.**
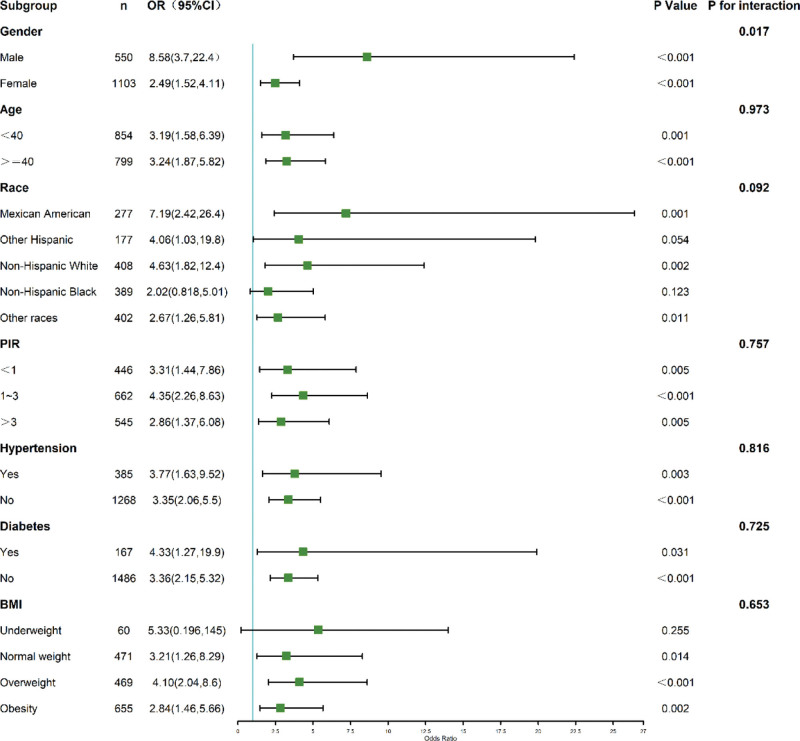
Subgroup analysis of testosterone (TST) and osteoporosis risk. Forest plot illustrating odds ratios (ORs) and 95% confidence intervals (CIs) for the association between testosterone levels and osteoporosis risk across demographic and clinical subgroups. Restricted cubic splines were used to model nonlinear relationships. Significant interactions are highlighted with asterisks (*P-interaction < .05*).

Subgroup results are presented in Figure 4 (for PLR) and Figure 5 (for TST). The positive association between high PLR (*Q*4 vs *Q*1) and osteoporosis was generally consistent across strata defined by age, sex, race/ethnicity, and BMI, with no significant interactions detected (all *P*-interaction > .05; Fig. 4). By contrast, the inverse association between TST and osteoporosis was (as expected) predominantly observed and much stronger in men than in women (*P*-interaction for sex < .001; Fig. 5). The association also appeared more pronounced among non-Hispanic Black participants and those with normal weight, although formal interaction tests for these strata were not statistically significant.

### 3.6. Sensitivity analysis in participants aged ≥50 years

Among participants aged ≥50 years (n = 712), association patterns paralleled those in the overall sample. Magnitudes were slightly attenuated but remained statistically significant for PLR *Q*4 versus *Q*1 (OR = 2.95, 95% CI: 1.45–6.01; *P* = .003) and for low TST among men (OR = 2.70, 95% CI: 1.55–4.70; *P* < .001). The PLR–TST interaction remained significant in this age group (*P*-interaction = .038), supporting the robustness of the main findings in the population for which the WHO osteoporosis definition is primarily validated.

## 4. Discussion

In this nationally representative study of U.S. adults, we found that higher PLR and lower TST levels were independently associated with increased prevalence of osteoporosis, and that these 2 factors interacted in a multiplicative manner. The association between PLR and osteoporosis was nonlinear, with a marked rise in risk above the upper quartile of PLR distribution. For TST, the relationship was nonlinear in men, with substantial increases in risk at very low levels, plateauing at intermediate concentrations, and potentially reversing at high levels. The interaction analysis indicated that co-occurrence of low TST and high PLR was associated with substantially greater osteoporosis odds than either factor alone.

Testosterone plays an essential role in bone metabolism regulation.^[24]^ Androgens directly influence bone homeostasis by acting on osteoblasts, osteoclasts, and osteocytes,^[25,26]^ and can also provide indirect protection via aromatization to estrogen.^[27]^ Our findings are in line with prior studies linking low TST to lower BMD and higher fracture risk.^[28–30]^ The spline analysis suggested a nonlinear relationship. However, given the relatively low number of osteoporosis cases (n ≈ 97) and the complexity of the models, the specific thresholds identified (e.g., ~22.08 and 33.34 ng/dL) should be interpreted as exploratory rather than definitive clinical cutoffs. For clinical interpretation in men, the established <300 ng/dL threshold remains the relevant benchmark.

PLR correlates with systemic inflammation and immune response status.^[31]^ Compared to other pro-inflammatory cytokines like interleukin-6 (IL-6), IL-9, and tumor necrosis factor-α (TNF-α), PLR is more accessible and stable.^[32]^ Inflammation may be involved in osteoporosis pathogenesis, characterized by oxidative stress and immune system activation, presenting a chronic state of systemic and subclinical inflammation.^[33,34]^ The correlation between PLR and osteoporosis has been widely discussed.^[35,36]^ Under inflammatory stimulation, excessive neutrophil activation releases reactive oxygen species and increases osteoclast production via the receptor activator of nuclear factor kappa-B ligand signaling pathway.^[37]^ Platelet-derived growth factors promote bone formation by influencing cell proliferation, chemotactic differentiation, and extracellular matrix synthesis, thereby playing a crucial role in bone homeostasis and remodeling. In reduced bone mass scenarios, platelet demand surpasses neutrophil demand, leading to increased PLR.

Many studies have explored the association between inflammation-related indicators and sex hormones.^[38]^ Testosterone, as the main androgen, has demonstrated anti-inflammatory and protective effects through various pathways and mechanisms. It reduces pro-inflammatory markers, including TNF-α, IL-6, IFN-γ, and IL-2, and inhibits nuclear factor kappa B and p65 signaling. Testosterone also reduces type 2 innate lymphoid cell proliferation and activity.^[39]^ These inflammatory components collectively participate in inflammatory disease pathogenesis.^[40]^ Conversely, inflammatory states and components affect TST production and secretion. Studies have confirmed that IL-6, TNF-α, and IL-1β impact the hypothalamic–pituitary–gonadal axis and testicular function. Similar to previous research, this study found significant correlations between TST levels, PLR, and osteoporosis occurrence, with a notable interaction between the 2, suggesting the existence of a “hormone-inflammation” dual pathway interaction in various physiological and pathological mechanisms.

Strengths of our study include the use of a large nationally representative dataset, standardized BMD assessment by dual-energy X-ray absorptiometry, reliable laboratory measures of TST and PLR, and advanced modeling techniques (RCS, interaction analysis). We adjusted for a comprehensive set of covariates, including demographic, socioeconomic, lifestyle, and comorbidity variables.

Several limitations should be acknowledged. First, the cross-sectional design precludes causal inference; associations observed cannot establish temporality. Second, the WHO definition of osteoporosis is primarily validated in adults ≥50 years; we addressed this limitation via sensitivity analysis restricted to this age group, which yielded similar results but cannot replace longitudinal validation. Third, nearly 88% of the initial NHANES sample was excluded due to missing data, raising the possibility of selection bias. Although we used weighted analyses to maintain population representation, this high exclusion rate may still impact the external validity of our findings, particularly if the excluded population differs systematically in bone health or hormonal status from the analytic sample. Fourth, some subgroup analyses (particularly among certain racial/ethnic categories and low BMI strata) were based on small numbers, limiting precision and generalizability. Fifth, single time-point measurements of TST and PLR may not reflect long-term levels.

Our results suggest that both inflammatory status and hormone levels should be considered jointly when assessing osteoporosis risk. Clinicians might consider evaluating inflammatory markers alongside sex hormones, especially in patients with risk factors for bone loss. Future studies should confirm these findings in longitudinal designs, explore underlying molecular mechanisms of hormone–inflammation interaction, and assess whether interventions targeting both pathways may yield additive benefits in preventing osteoporosis.

## 5. Conclusion

In a nationally representative sample of U.S. adults, higher PLR and lower serum TST levels were independently associated with greater odds of osteoporosis, and there was evidence of a significant multiplicative interaction between these 2 factors. These findings suggest that inflammatory burden and hormonal status may jointly contribute to osteoporosis risk. Further longitudinal and mechanistic studies are warranted to confirm these associations and to elucidate the biological basis of the hormone–inflammation interaction in bone metabolism.

## Acknowledgments

We appreciate all the NHANES participants and staff for their invaluable efforts and contributions.

## Author contributions

**Conceptualization:** Chenglu Tao, Huarui Shen, Ting Li.

**Data curation:** Chenglu Tao, Yingqi Liu.

**Formal analysis:** Yingqi Liu.

**Funding acquisition:** Yue He.

**Investigation:** Chenglu Tao, Yingqi Liu, Ting Li, Yue He.

**Methodology:** Chenglu Tao, Yingqi Liu, Huarui Shen.

**Project administration:** Huarui Shen, Ting Li.

**Resources:** Huarui Shen, Ting Li, Yue He.

**Software:** Huarui Shen, Ting Li, Yue He.

**Supervision:** Huarui Shen, Yue He.

**Validation:** Ting Li.

**Writing – original draft:** Chenglu Tao, Yingqi Liu.

**Writing – review & editing:** Chenglu Tao, Yingqi Liu.
